# Real-world prescription of anti-COVID-19 drugs in hospitalized patients with COVID-19 in Japan

**DOI:** 10.1371/journal.pone.0297679

**Published:** 2024-01-26

**Authors:** Haruka Shida, Maki Komamine, Kazuhiro Kajiyama, Takashi Waki, Hotaka Maruyama, Yoshiaki Uyama

**Affiliations:** Office of Medical Informatics and Epidemiology, Pharmaceuticals and Medical Devices Agency, Tokyo, Japan; Children’s National Hospital, George Washington University, UNITED STATES

## Abstract

**Objective:**

Prescription trends and patterns of anti-COVID-19 drugs in hospitalized patients were examined based on real world data to understand the use of anti-COVID-19 drugs in clinical practice in Japan.

**Design:**

The longitudinal and cross-sectional study was conducted utilizing data from January 1, 2019 to December 31, 2021 of the MID-NET^®^ medical information database, which stored the electronic medical records, administrative claim data, and diagnosis procedure combination data of patients in Japan.

**Participants:**

Hospitalized patients with a COVID-19-related diagnosis who received at least one anti-COVID-19 drug between April 1, 2020 and December 31, 2021.

**Exposures:**

The following 14 drugs were included in this study: remdesivir, baricitinib, combination product of casirivimab and imdevimab, favipiravir, dexamethasone, ivermectin, azithromycin, nafamostat mesylate, camostat mesylate, ciclesonide, tocilizumab, sarilumab, combination product of lopinavir and ritonavir, and hydroxychloroquine.

**Results:**

We identified 5,717 patients hospitalized with COVID-19 and prescribed at least one anti-COVID-19 drug. The entire cohort generally included patients over 41–50 years and more males. The most common prescription pattern was dexamethasone monotherapy (22.9%), followed by the concomitant use of remdesivir and dexamethasone (15.0%), azithromycin monotherapy (15.0%), remdesivir monotherapy (10.2%), and nafamostat mesylate monotherapy (8.5%). However, an often prescribed anti-COVID-19 drug differed depending on the period.

**Conclusions and relevance:**

This study revealed the real-world situation of anti-COVID-19 drug prescriptions in hospitalized COVID-19 patients in Japan. A prescribed drug would depend on the latest scientific evidence, such as efficacy, safety, and approval status, at the time of prescription. Understanding the prescription of anti-COVID-19 drugs will be important for providing the most up-to-date treatments to patients and evaluating the benefit and/or risk of anti-COVID-19 drugs based on the utilization of an electronic medical record database.

## Introduction

Coronavirus disease 2019 (COVID-19) is a rapidly evolving infectious/inflammatory disorder caused by severe acute respiratory syndrome coronavirus 2 (SARS-CoV-2) [1, 2]. Individuals infected with COVID-19 may experience the following stages of clinical features; an asymptomatic incubation period, the onset of disease with respiratory symptoms via local immune response, and disease progression with more severe disease symptoms through systemic inflammatory response such as cytokine-storm. Reportedly, controlling cytokine level by medical therapies is important for the management of COVID-19 patients including prevention of disease progression [1, 3].

In Japan, after the identification of the first case of coronavirus disease 2019 (COVID-19), various drugs have been used for its treatment, even with limited data available regarding the features of COVID-19 as well as its treatments by drugs [4]. With increasing scientific evidence and knowledge about COVID-19, the guidelines for its clinical treatment published by the Ministry of Health, Labour, and Welfare (MHLW), as well as by the academic society have also been updated in a short period to reflect the latest scientific knowledge [5–7]. Under such situations, understanding the real-world clinical practice of drug prescriptions for COVID-19 treatment, including changes in the temporal trend, is important for providing the most up-to-date treatments to patients and appropriately conducting benefit/risk assessments of drugs for COVID-19 treatment (anti-COVID-19 drugs). Therefore, in this study, we investigated real-world trends and prescription patterns of anti-COVID-19 drugs in hospitalized patients in Japan using the Medical Information Database Network (MID-NET^®^) [8] to accumulate data about the real-world use of anti-COVID-19 drugs in Japan.

## Methods

### Study design and setting

To examine the real-world trends and prescription patterns of anti-COVID-19 drugs in hospitalized patients in Japan, a longitudinal and cross-sectional study was conducted using MID-NET^®^, which is a reliable and valuable database of Japan [8, 9]. It stores electronic medical records, administrative claim data, and diagnosis procedure combination data of over 6.05 million patients (as of December 2022) in cooperation with 10 healthcare organizations, including 23 university hospitals and regional core hospitals. The study period spanned from January 1, 2019 (pre-period of the first patient with COVID-19 in Japan) to December 31, 2021 (the latest available data at the time this study commencement). MID-NET^®^ was selected as the data source for this study as it may more comprehensively cover prescription data for anti-COVID-19 drugs (i.e., also including drugs not covered by the national insurance) by analyzing multiple data categories, as described above. Additionally, a substantial number of moderate or severe patients who were hospitalized for the treatment of COVID-19 were expected in the cooperative hospitals of MID-NET^®^ [10].

The utilization of MID-NET^®^ for this study was approved on September 15, 2021 through a discussion at the expert committee of MID-NET^®^ [11] and actual data extraction from MID-NET^®^ for this study was conducted in the week of March 8, 2022. As this study was conducted as an official activity of the Pharmaceuticals and Medical Devices Agency (PMDA) under the Pharmaceuticals and Medical Devices Agency Law (Article 15–5–(c) and (f)), it was not subject to review by institutional review boards [12, 13] and the requirement for informed consent was waived. However, the opportunity of a patient to opt out from the MID-NET^® ^was ensured by all MID-NET^®^ cooperative hospitals.

### Target anti-COVID-19 drugs

Fourteen anti-COVID-19 drugs were determined as target drugs based on the clinical treatment guidelines published by the MHLW (version. 1.0–5.2, published by the end of study period) and the guideline on drug management for COVID-19 published by the Japanese Association for Infectious Disease (version 1.0–7.0, published by the end of study period) [5, 6]. These included remdesivir, baricitinib, combination product of casirivimab and imdevimab (casirivimab/imdevimab), favipiravir, dexamethasone, ivermectin, azithromycin, nafamostat mesylate, camostat mesylate, ciclesonide, tocilizumab, sarilumab, combination product of lopinavir and ritonavir, and hydroxychloroquine.

### Study population

The entire cohort comprised patients hospitalized with a COVID-19-related diagnosis who received at least one anti-COVID-19 drug during the investigation period between April 1, 2020 and December 31, 2021. Hospitalized patients were eligible based on the following inclusion criteria: 1) the COVID-19-related diagnosis codes were recorded within 14 days from the first prescription date of one of the 14 anti-COVID-19 drugs, and the earliest prescription date of the anti-COVID-19 drugs was set as t_0_.; 2) the additional charge for COVID-19-related hospitalization (t_1_) was recorded within 14 days from t_0_; 3) the COVID-19-related diagnosis codes were recorded within 14 days from t_1_; 4) the discharge date was recorded at least one day after t_0_. The investigation period began on April 1, 2020, because the additional charge specified for hospitalized patients with COVID-19 in the national claims system was implemented through a notice issued by the MHLW in April 2020 [14]. The COVID-19-related diagnosis was defined by the following International Classification of Diseases 10th revision (ICD-10) codes: U07.1 (including suspected codes), B34.2, B34.9, J18.0, J18.9, or J20.9. No exclusion criteria were applied in this study. The subcohorts were also created from the entire cohort with stratification for the prescription pattern of anti-COVID-19 drugs, including monotherapy and combination, at t_0_.

### Study measures

The number of patients prescribed each prescription pattern at t_0_ per month was counted from April 1, 2020 to December 31, 2021, and that of newly confirmed COVID-19 cases in Japan for the same period was collected from the national open data source published by the MHLW [15].

Patient characteristics at t_0_ were assessed based on the following factors: sex, age, year and month of prescription, comorbidities (chronic obstructive pulmonary disease, chronic kidney disease, diabetes mellitus, hypertension, hyperlipidemia, cardiovascular disease, cerebrovascular disease, bronchial asthma, malignant tumor, and hepatic disease), concomitant drugs (steroid, Janus kinase inhibitor, biologics, immunodepressant, angiotensin converting enzyme inhibitor, angiotensin II receptor blocker, statin, anticoagulant, and antiplatelets), blood purification therapy (plasma exchange, hemoadsorption, continuous hemofiltration, and cytapheresis), oxygen therapy (oxygen supplementation, non-invasive ventilation, and invasive ventilation, including extra-corporeal membrane oxygenation [ECMO]), additional charge specified for hospitalized patients with COVID-19 (mild-moderate, moderate or higher, and severe), and admission to the intensive care unit (ICU). Blood tests (biochemical tests; aspartate aminotransferase (AST), alanine aminotransferase (ALT), serum creatinine (Cr), and estimated glomerular filtration (eGFR) derived from the GFR estimation formula for the Japanese population [16]) and severity-related biomarkers (lymphocyte, D-dimer, C-reactive protein (CRP), lactate dehydrogenase (LDH), serum ferritin, troponin-I, and serum Krebs von den Lungen 6 (KL-6)) were assessed based on the results close to t_0_ during the period between the date of admission and t_0_. We selected liver function tests (AST and ALT) and kidney function tests (Cr and eGFR) as remdesivir had risks of liver function disorder and acute kidney injury at the time of special approval for emergency use as a COVID-19 treatment, and severity-related biomarkers based on the aforementioned guidelines and previous studies.

Changes in prescription patterns of anti-COVID-19 drugs in an individual patient from t_0_ to the early date of discharge date or end of study period were also assessed.

### Descriptive analysis

Patient characteristics were descriptively summarized for the entire cohort and subcohorts. Data were presented as a number and percentage for categorical variables or a median with the interquartile range (IQR) for continuous variables. Blood tests and severity-related biomarkers were also calculated for patients with abnormal results based on the thresholds reported in guidelines and previous studies [17–25]. The changes in prescription patterns of anti-COVID-19 drugs in an individual patient were visualized through Sankey diagrams. SAS 9.4 (SAS Institute, Cary, NC, USA) was used for all analyses.

## Results

### Patient characteristics

A total of 315,940 patients with COVID-19-related diagnosis were identified in this study. Among those, 5,717 patients were hospitalized with the prescription at least one anti-COVID-19 drug. The most common prescription pattern was dexamethasone monotherapy (22.9%), followed by the concomitant use of remdesivir and dexamethasone (remdesivir+dexamethasone, 15.0%), azithromycin monotherapy (15.0%), remdesivir monotherapy (10.2%), and nafamostat mesylate monotherapy (8.5%). Patient characteristics of the entire cohort and subcohorts for each prescription pattern with more than 50 patients are presented in Table 1. The entire cohort generally comprised patients over the age of 41–50 years, with a higher proportion of males (64.7%), with comorbidities, such as diabetes (17.1%) and hypertension (16.5%), and usually with anticoagulants (36.3%). The subcohorts for patients with dexamethasone monotherapy or its concomitant use with other drugs (remdesivir+dexamethason, remdesivir+dexamethason+baricitinib, and favipiravir+dexamethason) had a relatively higher percentage of oxygen therapy (63.8–91.6%), additional charge for hospitalized patients with COVID-19 (52.9–79.2% for moderate or higher severity), and admission to the ICU (28.5–57.0%). The percentage of admission to the ICU was also higher in the subcohorts for patients with remdesivir monotherapy (43.5%), favipiravir monotherapy (54.7%), or casirivimab/imdevimab (48.2%) than other subcohorts. Higher percentages of invasive ventilation for patients on remdesivir monotherapy (20.4%), and higher percentage of required oxygen in addition to invasive ventilation for patients treated with remdesivir+baricitinib (over 81.8% required oxygen therapy and 32.7% required invasive ventilation) were observed. Notably, patients with nafamostat mesylate monotherapy had more distinctive backgrounds, such as a higher percentage of blood purification therapy (53.0%).

**Table 1:** Patient characteristics of the entire cohort and subcohort at first prescription.

	ALL	Dexamethasone^b^	Remdesivir + Dexamethasone^b^	Azithromycin^b^	Remdesivir^b^	Nafamostat mesylate^b^	Favipiravir^b^
	N = 5,717	N = 1,311	N = 859	N = 857	N = 582	N = 485	N = 214
Age, n (%)							
0–10	99 (1.7)	68 (5.2)	0 (0.0)	18 (2.1)	< 10 (< 1.7)	< 10 (< 2.1)	0 (0.0)
11–20	43 (0.8)	11 (0.8)	< 10 (< 1.2)	10 (1.2)	< 10 (< 1.7)	< 10 (< 2.1)	< 10 (< 4.7)
21–30	225 (3.9)	38 (2.9)	< 30 (< 3.5)	33 (3.9)	37 (6.4)	14 (2.9)	< 20 (< 9.3)
31–40	340 (5.9)	70 (5.3)	60 (7.0)	39 (4.6)	49 (8.4)	16 (3.3)	16 (7.5)
41–50	770 (13.5)	157 (12.0)	172 (20.0)	60 (7.0)	97 (16.7)	30 (6.2)	29 (13.6)
51–60	1,059 (18.5)	221 (16.9)	227 (26.4)	75 (8.8)	131 (22.5)	63 (13.0)	40 (18.7)
61–70	957 (16.7)	222 (16.9)	159 (18.5)	113 (13.2)	101 (17.4)	81 (16.7)	42 (19.6)
71–80	1,234 (21.6)	298 (22.7)	121 (14.1)	235 (27.4)	108 (18.6)	151 (31.1)	49 (22.9)
>80	990 (17.3)	226 (17.2)	90 (10.5)	274 (32.0)	52 (8.9)	119 (24.5)	26 (12.1)
Sex, n (%)							
Male	3,701 (64.7)	842 (64.2)	594 (69.2)	543 (63.4)	380 (65.3)	313 (64.5)	126 (58.9)
Female	2,016 (35.3)	469 (35.8)	265 (30.8)	314 (36.6)	202 (34.7)	172 (35.5)	88 (41.1)
Comorbidities, n (%)							
Hypertension	944 (16.5)	175 (13.3)	171 (19.9)	120 (14.0)	131 (22.5)	59 (12.2)	25 (11.7)
Diabetes mellitus	978 (17.1)	158 (12.1)	170 (19.8)	83 (9.7)	207 (35.6)	44 (9.1)	24 (11.2)
Cardiovascular disease	596 (10.4)	75 (5.7)	77 (9.0)	94 (11.0)	148 (25.4)	59 (12.2)	17 (7.9)
Hyperlipidemia	497 (8.7)	79 (6.0)	97 (11.3)	58 (6.8)	80 (13.7)	30 (6.2)	13 (6.1)
Malignant tumor	282 (4.9)	41 (3.1)	28 (3.3)	27 (3.2)	121 (20.8)	16 (3.3)	< 10 (<4.7)
Bronchial asthma	334 (5.8)	80 (6.1)	42 (4.9)	44 (5.1)	39 (6.7)	< 10 (< 2.1)	10 (4.7)
Hepatic disease	222 (3.9)	37 (2.8)	24 (2.8)	17 (2.0)	16 (2.7)	26 (5.4)	< 10 (< 4.7)
Chronic kidney disease	172 (3.0)	20 (1.5)	12 (1.4)	20 (2.3)	19 (3.3)	57 (11.8)	< 10 (< 4.7)
Cerebrovascular disease	133 (2.3)	25 (1.9)	12 (1.4)	29 (3.4)	20 (3.4)	15 (3.1)	< 10 (< 4.7)
COPD	96 (1.7)	14 (1.1)	11 (1.3)	37 (4.3)	12 (2.1)	< 10 (< 2.1)	0 (0.0)
Concomitant drugs, n (%)							
Anticoagulant	2,076 (36.3)	464 (35.4)	598 (69.6)	131 (15.3)	224 (38.5)	141 (29.1)	43 (20.1)
Steroid excluding dexamethasone	746 (13.0)	62 (4.7)	18 (2.1)	173 (20.2)	102 (17.5)	96 (19.8)	20 (9.3)
Antiplatelet	569 (10.0)	70 (5.3)	38 (4.4)	57 (6.7)	35 (6.0)	45 (9.3)	23 (10.7)
ARB	387 (6.8)	69 (5.3)	70 (8.1)	53 (6.2)	48 (8.2)	19 (3.9)	19 (8.9)
Statin	366 (6.4)	64 (4.9)	58 (6.8)	53 (6.2)	48 (8.2)	34(7.0)	15 (7.0)
Janus kinase inhibitor	298 (5.2)	0 (0.0)	0 (0.0)	0 (0.0)	0 (0.0)	0 (0.0)	0 (0.0)
Biologics	163 (2.9)	0 (0.0)	0 (0.0)	0 (0.0)	0 (0.0)	0 (0.0)	0 (0.0)
Immunodepressant	53 (0.9)	10 (0.8)	< 10 (< 1.2)	14 (1.6)	< 10 (< 1.7)	< 10 (< 2.1)	< 10(< 4.7)
ACE	31 (0.5)	< 10 (< 0.8)	< 10 (< 1.2)	< 10 (< 1.2)	< 10 (< 1.7)	< 10 (< 2.1)	< 10 (< 4.7)
Blood purification therapy, n (%)	290 (5.1)	< 10 (< 0.8)	< 10 (< 1.2)	< 10 (< 1.2)	< 10 (< 1.7)	257 (53.0)	< 10 (< 4.7)
Oxygen therapy, n (%)							
No oxygen	1,914 (33.5)	474 (36.2)	113 (13.2)	346 (40.4)	234 (40.2)	158 (32.6)	85 (39.7)
Oxygen	3,803 (66.5)	837 (63.8)	746 (86.8)	511 (59.6)	348 (59.8)	327 (67.4)	129 (60.3)
Oxygen supplementation^a^	2,715 (47.5)	671 (51.2)	547 (63.7)	404 (47.1)	190 (32.6)	140 (28.9)	115 (53.7)
Non-invasive ventilation^a^	319 (5.6)	50 (3.8)	70 (8.1)	26 (3.0)	39 (6.7)	15 (3.1)	< 10 (< 4.7)
Invasive ventilation^a^	842 (14.7)	137 (10.5)	131 (15.3)	91 (10.6)	119 (20.4)	191 (39.4)	12 (5.6)
Additional charge specified for hospitalized patients with COVID-19, n (%)							
Mild or moderate^a^	1,821 (31.9)	418 (31.9)	135 (15.7)	381 (44.5)	233 (40.0)	298 (61.4)	62 (29.0)
Moderate or higher^a^	3,111 (54.4)	693 (52.9)	615 (71.6)	374 (43.6)	291 (50.0)	115 (23.7)	150 (70.1)
Severe^a^	1,987 (34.8)	427 (32.6)	543 (63.2)	128 (14.9)	268(46.0)	57 (11.8)	64 (29.9)
ICU admission, n (%)	1,693 (29.6)	374 (28.5)	482 (56.1)	90 (10.5)	253 (43.5)	38 (7.8)	117(54.7)
	Ciclesonide^b^	Casirivimab and imdevimab^b^	Remdesivir +Dexamethasone +Baricitinib^b^	Camostat mesylate^b^	Favipiravir +Dexamethasone^b^	Ivermectin^b^	Remdesivir +Baricitinib^b^
	N = 126	N = 110	N = 107	N = 97	N = 96	N = 61	N = 55
Age, n (%)							
0–10	< 10 (< 7.9)	0 (0.0)	0 (0.0)	0 (0.0)	0 (0.0)	0 (0.0)	0 (0.0)
11–20	< 10 (< 7.9)	< 10 (< 9.1)	< 10 (< 9.3)	< 10 (< 10.3)	0 (0.0)	0 (0.0)	0 (0.0)
21–30	< 10 (< 7.9)	< 10 (< 9.1)	< 10 (< 9.3)	0 (0.0)	0 (0.0)	17 (27.9)	0 (0.0)
31–40	16 (12.7)	13 (11.8)	< 10 (< 9.3)	< 10 (< 10.3)	< 10 (< 10.4)	< 10 (< 16.4)	< 10 (< 18.2)
41–50	15 (11.9)	34 (30.9)	30 (28.0)	< 10 (< 10.3)	< 20 (< 20.8)	< 10 (< 16.4)	12 (21.8)
51–60	19 (15.1)	30 (27.3)	20 (18.7)	< 10 (< 10.3)	15 (15.6)	13 (21.3)	20 (36.4)
61–70	18 (14.3)	< 10 (< 9.1)	19 (17.8)	14 (14.4)	18 (18.8)	< 10 (< 16.4)	< 10 (< 18.2)
71–80	25 (19.8)	< 10 (< 9.1)	15 (14.0)	30 (30.9)	23 (24.0)	< 10 (< 16.4)	< 10 (< 18.2)
>80	19 (15.1)	10 (9.1)	11 (10.3)	33 (34.0)	25 (26.0)	12 (19.7)	< 10 (< 18.2)
Sex, n (%)							
Male	66 (52.4)	65 (59.1)	65 (60.7)	64 (66.0)	68 (70.8)	37 (60.7)	42 (76.4)
Female	60 (47.6)	45 (40.9)	42 (39.3)	33 (34.0)	28 (29.2)	24 (39.3)	13 (23.6)
Comorbidities, n (%)							
Hypertension	20 (15.9)	13 (11.8)	21 (19.6)	11 (11.3)	14 (14.6)	< 10 (< 16.4)	13 (23.6)
Diabetes mellitus	17 (13.5)	< 10 (< 9.1)	36 (33.6)	< 10 (< 10.3)	20 (20.8)	10 (16.4)	22 (40.0)
Cardiovascular disease	< 10 (< 7.9)	< 10 (< 9.1)	15 (14.0)	< 10 (< 10.3)	12 (12.5)	10 (16.4)	< 10 (< 18.2)
Hyperlipidemia	11 (8.7)	< 10 (< 9.1)	16 (15.0)	< 10 (< 10.3)	10 (10.4)	< 10 (< 16.4)	< 10 (< 18.2)
Malignant tumor	< 10 (< 7.9)	< 10 (< 9.1)	< 10 (< 9.3)	< 10 (< 10.3)	< 10 (< 10.4)	< 10 (< 16.4)	< 10 (< 18.2)
Bronchial asthma	14 (11.1)	< 10 (< 9.1)	< 10 (< 9.3)	< 10 (< 10.3)	< 10 (< 10.4)	< 10 (< 16.4)	< 10 (< 18.2)
Hepatic disease	< 10 (< 7.9)	< 10 (< 9.1)	< 10 (< 9.3)	< 10 (< 10.3)	< 10 (< 10.4)	0 (0.0)	< 10 (< 18.2)
Chronic kidney disease	< 10 (< 7.9)	< 10 (< 9.1)	0 (0.0)	< 10 (< 10.3)	< 10 (< 10.4)	< 10 (< 16.4)	0 (0.0)
Cerebrovascular disease	< 10 (< 7.9)	0 (0.0)	0 (0.0)	< 10 (< 10.3)	< 10 (< 10.4)	0 (0.0)	< 10 (< 18.2)
COPD	< 10 (< 7.9)	< 10 (< 9.1)	0 (0.0)	0 (0.0)	< 10 (< 10.4)	0 (0.0)	0 (0.0)
Concomitant drugs, n (%)							
Anticoagulant	< 10 (< 7.9)	10 (9.1)	83 (77.6)	15 (15.5)	26 (27.1)	< 10 (< 16.4)	40 (72.7)
Steroid excluding dexamethasone	20 (15.9)	< 10 (< 9.1)	< 10 (< 9.3)	< 10 (< 10.3)	< 10 (< 10.4)	0 (0.0)	42 (76.4)
Antiplatelet	28 (22.2)	< 10 (< 9.1)	< 10 (< 9.3)	15 (15.5)	12 (12.5)	< 10 (< 16.4)	< 10 (< 18.2)
ARB	< 10 (< 7.9)	< 10 (< 9.1)	10 (9.3)	17 (17.5)	11 (11.5)	< 10 (< 16.4)	< 10 (< 18.2)
Statin	< 10 (< 7.9)	< 10 (< 9.1)	12 (11.2)	15 (15.5)	14 (14.6)	< 10 (< 16.4)	< 10 (< 18.2)
Janus kinase inhibitor	0 (0.0)	0 (0.0)	107 (100.0)	0 (0.0)	0 (0.0)	0 (0.0)	55 (100.0)
Biologics	0 (0.0)	0 (0.0)	0 (0.0)	0 (0.0)	0 (0.0)	0 (0.0)	0 (0.0)
Immunodepressant	0 (0.0)	< 10 (< 9.1)	< 10 (< 9.3)	< 10 (< 10.3)	0 (0.0)	< 10 (< 16.4)	< 10 (< 18.2)
ACE	0 (0.0)	0 (0.0)	0 (0.0)	< 10 (< 10.3)	0 (0.0)	< 10 (< 16.4)	0 (0.0)
Blood purification therapy, n (%)	0 (0.0)	0 (0.0)	0 (0.0)	0 (0.0)	0 (0.0)	0 (0.0)	0 (0.0)
Oxygen therapy, n (%)							
No oxygen	90 (71.4)	95 (86.4)	< 10 (< 9.3)	71 (73.2)	17 (17.7)	46 (75.4)	< 10 (< 18.2)
Oxygen	36 (28.6)	15 (13.6)	< 100 (< 93.5)	26 (26.8)	79 (82.3)	15 (24.6)	> 45 (> 81.8)
Oxygen supplementation^a^	35 (27.8)	15 (13.6)	88 (82.2)	24 (24.7)	76 (79.2)	15 (24.6)	21 (38.2)
Non-invasive ventilation^a^	< 10 (< 7.9)	0 (0.0)	< 10 (< 9.3)	0 (0.0)	< 10 (< 10.4)	0 (0.0)	19 (34.5)
Invasive ventilation^a^	0 (0.0)	0 (0.0)	< 10 (< 9.3)	< 10 (< 10.3)	< 10 (< 10.4)	0 (0.0)	18 (32.7)
Additional charge specified for hospitalized patients with COVID-19, n (%)							
Mild or moderate^a^	39 (31.0)	16 (14.5)	19 (17.8)	58 (59.8)	14 (14.6)	41 (67.2)	< 10 (< 18.2)
Moderate or higher^a^	82 (65.1)	14 (12.7)	82 (76.6)	15 (15.5)	76 (79.2)	18 (29.5)	36 (65.5)
Severe^a^	< 10 (< 7.9)	82 (74.5)	21 (19.6)	< 10 (< 10.3)	37 (38.5)	0 (0.0)	26 (47.3)
ICU admission, n (%)	< 10 (< 7.9)	53 (48.2)	61 (57.0)	< 10 (< 10.3)	38 (39.6)	0 (0.0)	13 (23.6)

ACE, angiotensin converting enzyme inhibitor; ARB, angiotensin II receptor blocker; COPD, chronic obstructive pulmonary disease; ICU, intensive care unit.

Data for subcohorts for each prescription pattern with more than 50 patients are presented.

^a^ When multiple procedures were identified in a patient at first prescription, they were separately counted.

^b^ When a value was less than 10, it was indicated as an aggregated value based on the MID-NET^®^ publication rule.

The results of blood tests and severity-related biomarkers are presented in Table 2 and S1 Table. The median values of blood tests (AST, ALT, Cr, and eGFR) in the entire cohort were almost within the normal range, whereas some abnormal median values were observed in the subcohorts for patients with nafamostat mesylate monotherapy. The median values of the biomarkers related to the severity of COVID-19 (lymphocyte, D-dimer, CRP, LDH, serum ferritin, troponin-I, and KL-6), in the entire cohort were almost outside the normal range.

**Table 2:** Baseline characteristics of patients on laboratory test results.

	ALL	Dexamethasone	Remdesivir + Dexamethasone	Azithromycin	Remdesivir	Nafamostat mesylate	Favipiravir
	N = 5,717	N = 1,311	N = 859	N = 857	N = 582	N = 485	N = 214
**Blood tests (biochemical tests), median (IQR)**							
AST, IU/L	38.0 (25.0–60.0)	38.0 (26.0–60.0)	47.0 (34.0–71.0)	28.0 (20.0–42.0)	37.0 (26.0–54.0)	51.3 (23.0–143.0)	30.0 (23.0–45.0)
ALT, IU/L	28.0 (17.0–50.0)	28.0 (17.0–49.0)	37.0 (24.0–60.0)	20.0 (13.0–33.0)	28.0 (18.0–48.0)	31.0 (14.0–92.0)	25.0 (15.0–42.0)
Serum creatinine, mg/dL	0.87 (0.68–1.13)	0.83 (0.65–1.04)	0.86 (0.69–1.06)	0.84 (0.67–1.12)	0.82 (0.66–1.05)	2.87 (1.03–5.58)	0.83 (0.67–1.07)
eGFR, mL/min/1.73m^2^	64.8 (47.4–80.8)	66.2 (50.8–82.9)	68.4 (53.2–80.8)	63.5 (46.7–81.9)	70.3 (54.0–84.7)	16.9 (7.7–52.7)	65.7 (50.0–80.3)
**Severity-related biomarkers, median (IQR)**							
Lymphocytes, %	13.8 (8.1–21.5)	15.2 (9.3–22.7)	14.1 (9.1–20.5)	10.0 (6.2–16.2)	15.5 (8.2–24.0)	8.1 (5.0–14.5)	19.0 (12.7–26.8)
D-dimer, μg/mL	1.2 (0.7–2.6)	1.3 (0.8–2.9)	1.0 (0.6–1.6)	2.0 (1.0–4.3)	1.0 (0.5–1.8)	6.0 (2.2–13.9)	0.8 (0.5–1.3)
CRP, mg/dL	6.1 (2.2–11.8)	5.2 (1.6–10.4)	7.7 (4.3–12.6)	8.8 (3.5–15.9)	5.0 (2.2–9.1)	7.1 (1.1–15.6)	3.2 (1.0–7.3)
LDH, U/L	317.0 (233.0–446.0)	320.5 (233.0–440.0)	399.0 (304.0–513.0)	256.0 (202.0–330.0)	316.0(243.0–452.0)	325.8 (229.0–529.0)	254.0 (204.0–347.0)
Serum ferritin, ng/mL	497.9 (218.7–983.4)	482.0 (211.0–956.0)	782.9 (399.2–1300.0)	242.7 (111.2–461.8)	463.0 (217.0–925.0)	373.0 (133.0–840.0)	350.0 (151.6–636.4)
Troponin-I, ng/mL	0.01 (0.01–0.04)	0.01 (0.01–0.03)	0.01 (0.01–0.02)	0.03 (0.01–0.11)	0.01 (0.01–0.02)	0.13 (0.03–1.65)	0.01 (0.01–0.01)
KL-6, U/mL	270.0 (199.0–432.0)	287.0 (212.5–437.5)	267.0 (201.0–400.0)	401.0 (227.0–822.8)	269.0 (194.4–449.0)	235.5 (192.5–388.0)	215.0 (172.0–299.0)
	Ciclesonide	Casirivimab and imdevimab	Remdesivir +Dexamethasone + Baricitinib	Camostat mesylate	Favipiravir + Dexamethasone	Ivermectin	Remdesivir + Baricitinib
	N = 126	N = 110	N = 107	N = 97	N = 96	N = 61	N = 55
**Blood tests (biochemical tests), median, (IQR)**							
AST, IU/L	27.0 (20.0–39.0)	28.0 (21.0–41.0)	46.0 (34.0–66.5)	28.0 (21.0–42.0)	38.5 (29.0–55.3)	22.0 (17.0–31.0)	66.0 (46.0–79.0)
ALT, IU/L	20.5 (14.0–34.0)	22.0 (17.0–43.0)	33.0 (23.0–57.5)	19.5 (12.0–47.0)	29.3 (18.5–48.0)	18.0 (12.0–26.0)	50.0 (31.0–64.0)
Serum creatinine, mg/dL	0.81 (0.61–0.98)	0.87 (0.73–1.05)	0.82 (0.65–0.96)	0.82 (0.67–1.17)	0.94 (0.79–1.15)	0.88 (0.72–1.01)	0.88 (0.70–1.06)
eGFR, mL/min/1.73m^2^	68.5 (56.8–86.0)	67.6 (58.0–77.0)	72.9 (57.2–86.4)	65.7 (46.5–83.1)	59.7 (47.7–68.3)	71.4 (60.1–82.2)	68.4 (54.3–84.4)
**Severity-related biomarkers, median, (IQR)**							
Lymphocytes, %	21.4 (14.5–27.0)	20.9 (13.5–29.0)	13.8 (9.1–21.0)	17.3 (9.0–23.6)	13.8 (9.6–19.2)	25.9 (21.0–34.6)	11.2 (9.2–16.3)
D-dimer, μg/mL	0.7 (0.5–1.2)	0.5 (0.5–0.8)	1.0 (0.7–1.4)	1.7 (0.9–4.1)	1.1 (0.8–1.8)	0.7 (0.5–1.1)	1.4 (1.2–1.8)
CRP, mg/dL	2.3 (0.5–6.0)	1.5 (0.6–3.8)	8.4 (4.2–12.4)	2.4 (0.8–5.9)	6.5 (3.9–11.3)	1.0 (0.1–3.4)	10.4 (5.0–15.1)
LDH, U/L	234.5 (183.0–316.0)	218.0 (173.0–288.0)	404.0 (342.5–488.5)	221.0 (176.0–289.0)	315.0 (244.0–426.0)	205.0 (179.0–261.0)	499.5 (401.0–590.0)
Serum ferritin, ng/mL	222.3 (107.3–476.3)	226.5 (118.0–514.9)	697.0 (403.0–1302.0)	168.0 (126.0–511.0)	499.8 (285.0–788.0)	96.0 (51.0–255.0)	217.6 (761.0–1898.0)
Troponin-I, ng/mL	0.01 (0.01–0.02)	0.01 (0.00–0.02)	0.01 (0.01–0.01)	0.03 (0.01–0.05)	0.01 (0.01–0.02)	0.02 (0.01–0.17)	0.01 (0.01–0.02)
KL-6, U/mL	230.0 (174.0–307.0)	223.5 (176.0–309.2)	246.8 (190.1–369.6)	233.4 (188.0–307.0)	258.5 (190.0–412.1)	203.0 (155.0–236.0)	348.5 (247.0–433.0)

AST, aspartate aminotransferase; ALT, alanine aminotransferase; CRP, C-reactive protein; eGFR, estimated glomerular filtration; IQR, interquartile range; KL-6, serum Krebs von den Lungen 6; LHD, lactate dehydrogenase.

* The number of patients with the results of laboratory test was reported in S1 Table.

### Prescription pattern and its trend

**Fig 1:**
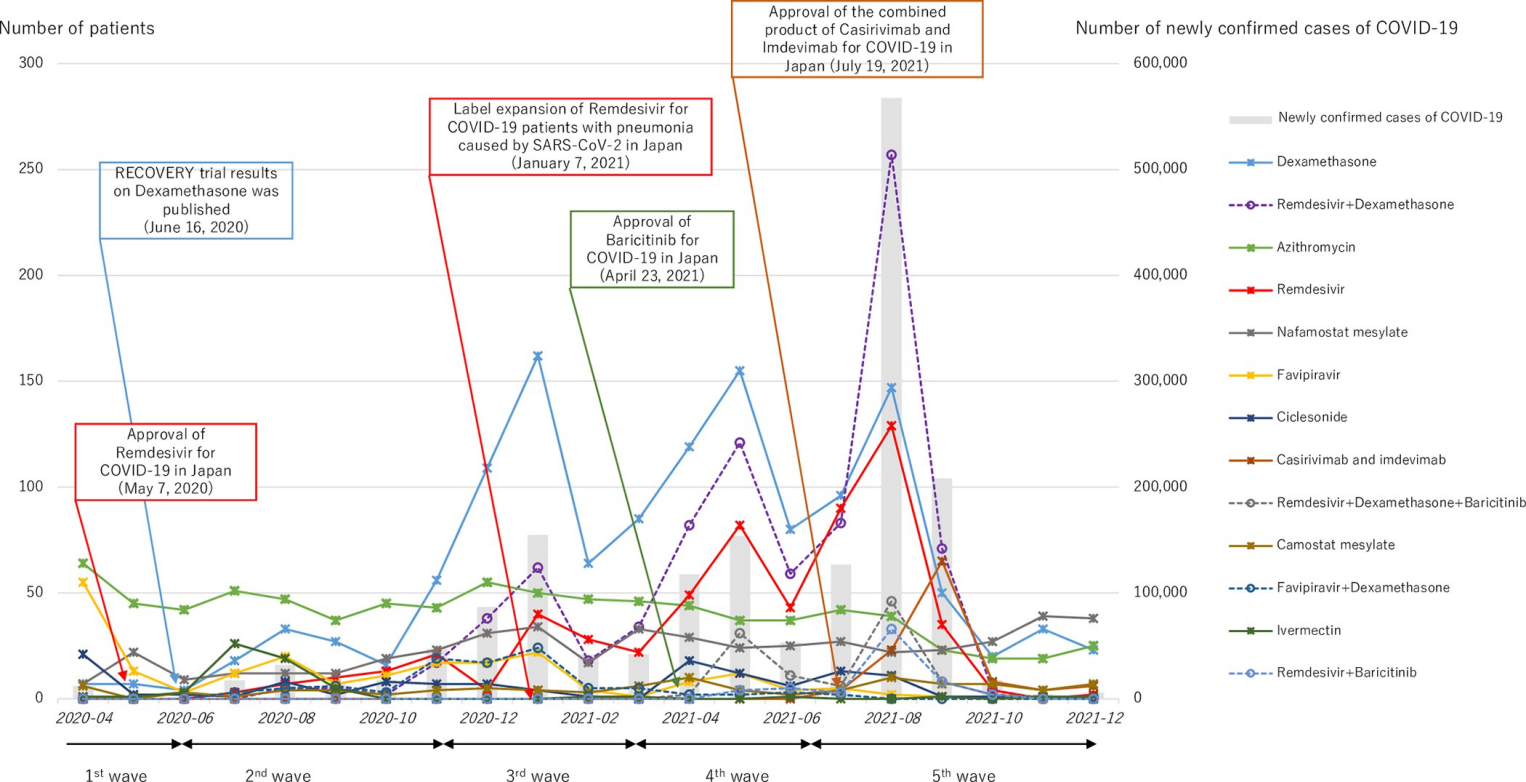
Trend of prescription pattern of anti-COVID-19 drugs in 2020 and 2021 (first to fifth waves).

The trend of prescription patterns of anti-COVID-19 drugs during the investigation period is shown in Fig 1. This period covered the first to fifth waves of COVID-19 in Japan, and the number of hospitalized patients prescribed anti-COVID-19 drugs increased or decreased in accordance with the number of newly confirmed cases of COVID-19 in Japan. Notably, azithromycin and favipiravir were the major prescribed drugs at the beginning of the investigation period, but the number of patients prescribed these drugs gradually decreased. Conversely, the number of patients with remdesivir or dexamethasone was relatively small in the early period but gradually increased after July 2020. In August 2021, when the highest number of newly confirmed cases of COVID-19 were observed, remdesivir+dexamethasone, dexamethasone monotherapy, or remdesivir monotherapy accounted for the majority of prescription. The number of patients with nafamostat monotherapy was relatively stable with some fluctuation associated with the newly confirmed COVID-19 cases in Japan. The number of patients on camostat mesylate or ivermectin monotherapies was relatively small during investigation period. The number of prescriptions for more recently approved drugs, such as baricitinib and casirivimab/imdevimab, was limited but increased in the fifth wave.

### Changes in prescription patterns of anti-COVID-19 drugs in an individual patient

**Fig 2:**
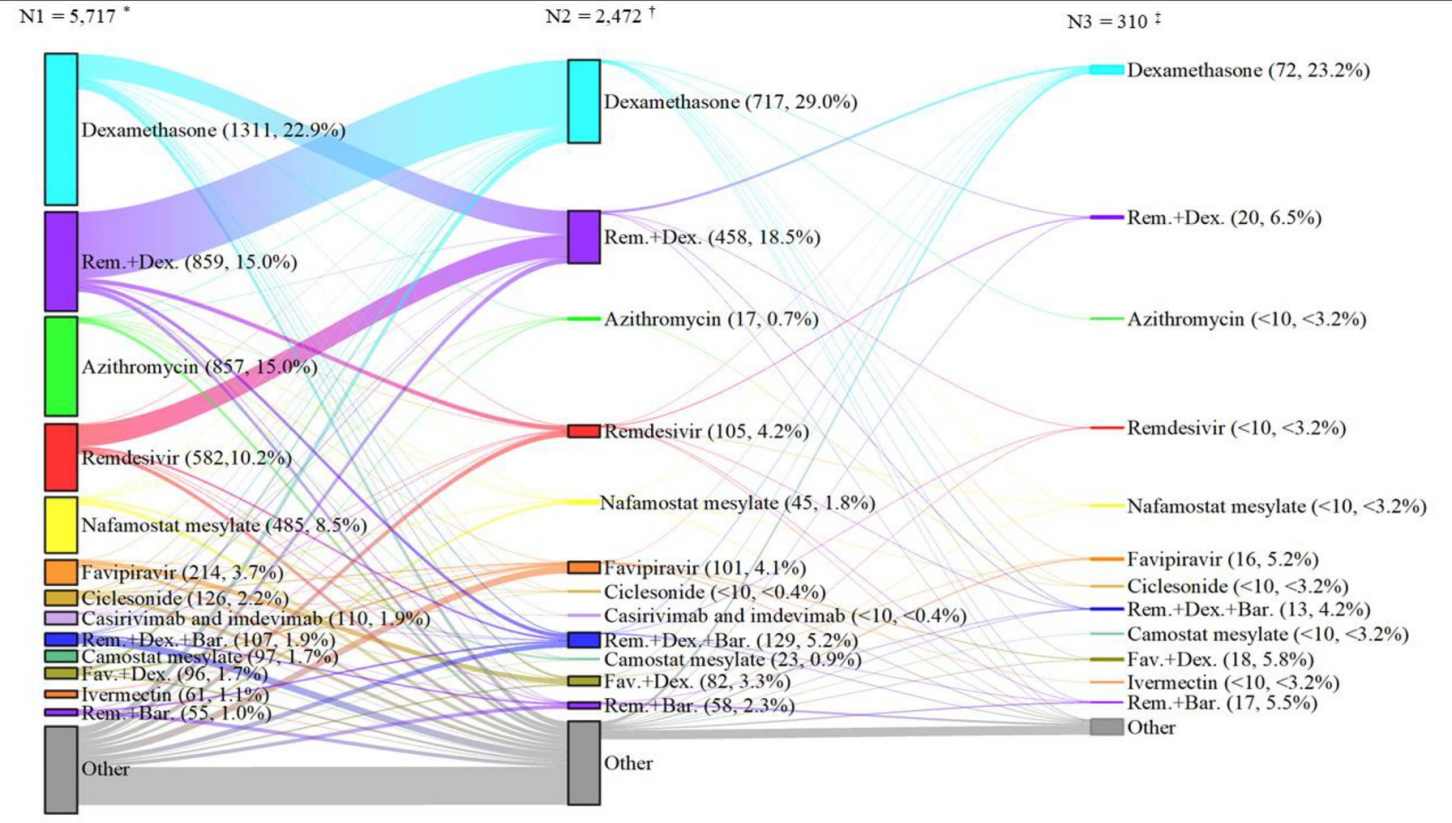
Change in prescription patterns of anti-COVID-19 drugs in an individual patient. Bar, Baricitinib; Dex, dexamethasone; Fav, Favipiravir; Rem, remdesivir. “Other” represents the other prescription patterns. * N1 indicates the number of hospitalized patients with COVID-19 who received at least one anti-COVID-19 drug (entire cohort). The percentage represent the proportion of the number of patients prescribed each prescription pattern to N1. ^†, ‡^ N2 and N3 indicate the number of patients who have changed to subsequent prescribed patterns. The percentage represent the proportion of the number of patients prescribed each prescription pattern to N2 or N3.

As shown in Fig 2, the prescribed drugs were usually changed in a patient during hospitalization. Many patients who started with monotherapy of remdesivir (45.0%) or dexamethasone (23.6%) were switched to other therapies during their hospitalization, whereas a portion of patients with monotherapy of remdesivir (32.6%) or dexamethasone (15.6%) were switched to remdesivir+dexamethasone. Moreover, over half the patients (67.1%) who started with remdesivir+dexamethasone were subsequently switched to dexamethasone monotherapy.

## Discussion

Our study revealed the real-world situation of anti-COVID-19 drug prescriptions in hospitalized patients with COVID-19 in Japan. The number of patients prescribed anti-COVID-19 drugs were associated with the number of newly confirmed cases of COVID-19 in Japan [15]. Compared with those in the previous reports of the COVID-19 Registry Japan (COVIREGI-JP) [4], the general characteristics of patients enrolled in this study, such as the age distribution, increased male patients, and relatively higher percentage of diabetes and hypertension as comorbidities, were similar, but the percentages of patients requiring oxygen therapy and admission to the ICU were higher in this study. These differences could be ascribed to the characteristics of MID-NET^®^ cooperative hospitals that were mainly university hospitals and regional core hospitals [8]. These results suggest that data extracted from MID-NET^®^ appropriately reflected the real-world situation of clinical practice in Japan, although patients with more severe conditions of COVID-19 were included in this study.

The most frequently prescription pattern during the investigation period was dexamethasone monotherapy. The number of patients with dexamethasone was relatively small at the beginning but increased after the second wave. This could have been triggered by the publication of the Randomized Evaluation of COVID-19 Therapy (RECOVERY) trial results, designed to evaluate the effects of potential treatments in patients hospitalized with COVID-19 at 176 National Health Service organizations in the United Kingdom, indicating that treatment with dexamethasone reduced deaths in hospitalized COVID-19 patients in June 2020 [26]. The COVID-19 treatment guideline published by the National Institute of Health in the United States of America was revised to the use of dexamethasone for patients with COVID-19 requiring oxygen therapy in June 2020 [27]. Similarly in Japan, the clinical treatment guideline was revised in July 2020 to include the same recommendation [28]. These situations would reflect the backgrounds of patients prescribed dexamethasone or its concomitant use with other drugs, resulting in a higher percentage of patients requiring oxygen therapy and admission to the ICU.

The number of patients with remdesivir was relatively small even after the special approval of this drug for emergency use in May 2020 in Japan [29], but gradually increased after the label expansion for the patients with pneumonia caused by SARS-CoV-2 in January 2021 [30]. Further clarification of target patients of remdesivir stating expeditious administration for patients requiring oxygen supplementation, but not for patients requiring ventilation in the clinical guideline reported in December 2020, may have also affected the prescription of this drug [31].

Nafamostat mesylate might be prescribed for blood purification therapy and/or ECMO because of the anticoagulant effect of this drug [32]. Moreover, nafamostat mesylate may be prescribed for anticipating the inhibitory effect of the entry of SARS-CoV-2 into the human epithelial cells [33, 34]. The higher percentage of admission to the ICU for patients with nafamostat mesylate might be attributed to the blood purification therapy for COVID-19 in the early stage of multiple organ dysfunction syndrome [5]. Moreover, reports have stated that many patients prescribed nafamostat mesylate concomitantly used other anti-COVID-19 drugs in Japan [35]. Considering such situations, some patients prescribed nafamostat mesylate in this study may have required blood purification therapy only at the time of first prescription of nafamostat mesylate, but were later co-prescribed other anti-COVID-19 drugs. These unique characteristics may explain why the number of patients with nafamostat mesylate was relatively stable during the investigation period.

Favipiravir monotherapy was commonly prescribed at the beginning but gradually decreased in the later period. This may be attributed to the negative results (no statistical significances on the efficacy) in the Japanese clinical trial published in November 2020 [36]; although the earlier version of the guideline on drug management for COVID-19 (version 1.0) mentioned favipiravir as a candidate drug for the treatment of COVID-19 [37]. Similarly, the lack of improvement regarding the survival or other prespecified clinical outcomes among hospitalized COVID-19 patients by the use of azithromycin, reported in the RECOVERY trial in February 2021 [38], may have contributed to the gradual decrease of azithromycin prescription in the later period. However, azithromycin monotherapy was a common prescription pattern at the beginning and may have been used to counteract the immunomodulatory effects of COVID-19 in addition to its macrolide antibiotic effects [39–41].

Negative results on the efficacy in clinical trial or meta-analysis may also explain the small number of prescriptions for ciclesonide [42], ivermectin [43], and camostat mesylate [44].

The relatively small number of patients prescribed the newly approved anti-COVID-19 drugs, such as baricitinib [45] and casirivimab/imdevimab [46], in the later period could be attributed to the short investigation period of this study after drug approval in Japan. Their prescriptions may have increased in the next wave of COVID-19 because of the increased scientific evidence in clinical trials.

Moreover, the percentage of patients with ventilation did not always correlate with that of hospitalized patients in the ICU. For example, in patients with nafamostat mesylate, the percentage of hospitalization in the ICU was lower (7.8%) but was higher for invasive ventilation (39.4%). Conversely, in patients with casirivimab/imdevimab or remdesivir+dexamethasone+baricitinib, the percentage of hospitalization in the ICU was higher (48.2 or 57.0%) but was lower for invasive ventilation (0.0 or 3.7%). These may depend on the number of newly infected COVID-19 patients and the availability of ICU beds in hospitals during the pandemic.

As described above, the most frequently prescribed anti-COVID-19 drugs varied depending on the period and generally coincided with available data regarding their efficacy, safety, and approval status. This indicated that during the clinical practice, an anti-COVID-19 drug was prescribed to a patient based on the latest scientific evidence at the time of prescription. Additionally, the prescription of a patient was changed during hospitalization based on the available scientific evidence and the patient’s severity [5, 6].

The results of this study enhance our understanding how anti-COVID-19 drugs are prescribed in accordance with scientific advances, and facilitate correct understanding of the prescription of anti-COVID-19 drugs in a real-world situation during the pandemic. Such knowledge will be important for not only considering the drug to be prescribed in clinical practice but also for more appropriately planning a pharmaco-epidemiological study to evaluate the benefit and/or risk of anti-COVID-19 drugs based on the secondary utilization of electronic medical record databases, such as MID-NET^®^.

Under situations of insufficient data regarding the efficacy and safety of anti-COVID-19 drugs at the time of drug approval, and when scientific evidence has been rapidly gathered in a short period after approval, an expedite evaluation of drug efficacy and safety in clinical practice based on real-world data will be more important. This is because conditions and available therapies could dramatically differ from the situation at the time of drug approval. The PMDA will continue to conduct benefit/risk assessments of anti-COVID-19 drugs based on real-world data for ensuring the balance between benefit and risk in clinical practice.

### Limitations

This study has several limitations. First, as the recommendation standard for hospitalization changed during the study period and the place of hospitalization was coordinated by the regional health center, the representation of patients in this study may differ in the study period. Second, the prescription patterns might have been influenced by the available drugs and investigational clinical studies conducted in each hospital.

## Conclusions

Our study provides useful information about the real-world situation of anti-COVID-19 drug prescriptions in hospitalized COVID-19 patients in Japan. A prescribed drug in clinical practice depended on the latest scientific evidence, such as efficacy, safety, and approval status, at the time of prescription. Understanding how anti-COVID-19 drugs might be prescribed in line with scientific evidence is important for not only considering the drug to be prescribed in clinical practice but also for more appropriately planning a pharmaco-epidemiological study to evaluate the benefit and/or risk of anti-COVID-19 drugs based on the secondary utilization of electronic medical record databases.

## Supporting information

S1 Table The percentage of patients with abnormal laboratory test results. (DOCX)
