# Supplementary material for: Real-world prescription of anti-COVID-19 drugs in hospitalized patients with COVID-19 in Japan
Source: PLoS One. 2024 Jan 26;19(1):e0297679. doi: 10.1371/journal.pone.0297679 (PMC10817178; doi:10.1371/journal.pone.0297679)
Supplement: S1 Table — (DOCX) [file pone.0297679.s001.docx]

S1 Table. The percentage of patients with abnormal laboratory test results.

|  | ALL | Dexamethasone | Remdesivir + Dexamethasone | Azithromycin | Remdesivir | Nafamostat mesylate | Favipiravir |
| --- | --- | --- | --- | --- | --- | --- | --- |
|  | N = 5,717 | N = 1,311 | N = 859 | N = 857 | N = 582 | N = 485 | N = 214 |
| **Blood tests (biochemical tests)** | | | | | | | |
| AST | (n = 5,540) | (n = 1,275) | (n = 851) | (n = 821) | (n = 577) | (n = 478) | (n = 199) |
| Median, (IQR), IU/L | 38.0 (25.0-60.0) | 38.0 (26.0-60.0) | 47.0 (34.0-71.0) | 28.0 (20.0-42.0) | 37.0 (26.0-54.0) | 51.3 (23.0-143.0) | 30.0 (23.0-45.0) |
| ≥ 50, IU/L ^a^ | 1,883 (34.0) | 433 (33.9) | 392 (46.1) | 148 (18.0) | 177 (30.7) | 244 (51.0) | 39 (19.6) |
| ALT | (n = 5,545) | (n = 1,275) | (n = 851) | (n = 822) | (n = 579) | (n = 478) | (n = 199) |
| Median, (IQR), IU/L | 28.0 (17.0-50.0) | 28.0 (17.0-49.0) | 37.0 (24.0-60.0) | 20.0 (13.0-33.0) | 28.0 (18.0-48.0) | 31.0 (14.0-92.0) | 25.0 (15.0-42.0) |
| ≥ 50, IU/L ^a^ | 1,407 (25.4) | 315 (24.7) | 290 (34.1) | 105 (12.8) | 136 (23.5) | 176 (36.8) | 37 (18.6) |
| Serum creatinine | (n = 5,545) | (n = 1,275) | (n = 850) | (n = 822) | (n = 579) | (n = 478) | (n = 199) |
| Median, (IQR), mg/dL | 0.87 (0.68-1.13) | 0.83 (0.65-1.04) | 0.86 (0.69-1.06) | 0.84 (0.67-1.12) | 0.82 (0.66-1.05) | 2.87 (1.03-5.58) | 0.83 (0.67-1.07) |
| > 2 mg/dL ^a^ | 242 (4.4) | 21 (1.6) | 10 (1.2) | 18 (2.2) | 15 (2.6) | 146 (30.5) | < 10 ^b^ (< 5.0) |
| eGFR | (n = 5,517) | (n = 1,250) | (n = 850) | (n = 821) | (n = 579) | (n = 477) | (n = 199) |
| Median, (IQR), mL/min/1.73m^2^ | 64.8 (47.4-80.8) | 66.2 (50.8-82.9) | 68.4 (53.2-80.8) | 63.5 (46.7-81.9) | 70.3 (54.0-84.7) | 16.9 (7.7-52.7) | 65.7 (50.0-80.3) |
| ≤ 60, mL/min/1.73m^2 a^ | 4,635 (84.0) | 1,025 (82.0) | 727 (85.5) | 680 (82.8) | 469 (81.0) | 438 (91.8) | 170 (85.4) |
| **Severity-related biomarkers** | | | | | | | |
| Lymphocytes | (n = 5,334) | (n = 1,245) | (n = 836) | (n = 787) | (n = 535) | (n = 457) | (n = 191) |
| Median (IQR), % | 13.8 (8.1-21.5) | 15.2 (9.3-22.7) | 14.1 (9.1-20.5) | 10.0 (6.2-16.2) | 15.5 (8.2-24.0) | 8.1 (5.0-14.5) | 19.0 (12.7-26.8) |
| < 50% ^a^ | 5,287 (99.1) | 1224 (98.3) | 835 (99.9) | 779 (99.0) | 532 (99.4) | 452 (98.9) | 191 (100.0) |
| D-dimer | (n = 4,677) | (n = 1,088) | (n = 835) | (n = 451) | (n = 566) | (n = 335) | (n = 180) |
| Median (IQR), µg/mL | 1.2 (0.7-2.6) | 1.3 (0.8-2.9) | 1.0 (0.6-1.6) | 2.0 (1.0-4.3) | 1.0 (0.5-1.8) | 6.0 (2.2-13.9) | 0.8 (0.5-1.3) |
| > 1.5 µg/mL ^a^ | 1839 (39.3) | 463 (42.6) | 228 (27.3) | 268 (59.4) | 171 (30.2) | 272 (81.2) | 41 (22.8) |
| CRP | (n = 5,533) | (n = 1,264) | (n = 851) | (n = 821) | (n = 579) | (n = 477) | (n = 199) |
| Median (IQR), mg/dL | 6.1 (2.2-11.8) | 5.2 (1.6-10.4) | 7.7 (4.3-12.6) | 8.8 (3.5-15.9) | 5.0 (2.2-9.1) | 7.1 (1.1-15.6) | 3.2 (1.0-7.3) |
| 1-4 mg/dL ^a^ | 1,233 (22.3) | 306 (24.2) | 165 (19.4) | 135 (16.4) | 174 (30.1) | 68 (14.3) | 64 (32.2) |
| > 4 mg/dL ^a^ | 3,486 (63.0) | 724 (57.3) | 653 (76.7) | 592 (72.1) | 336 (58.0) | 292 (61.2) | 86 (43.2) |
| LDH | (n = 5,516) | (n = 1,266) | (n = 846) | (n = 819) | (n = 575) | (n = 478) | (n = 198) |
| Median (IQR), U/L | 317.0 (233.0-446.0) | 320.5 (233.0-440.0) | 399.0 (304.0-513.0) | 256.0 (202.0-330.0) | 316.0(243.0-452.0) | 325.8 (229.0-529.0) | 254.0 (204.0-347.0) |
| > 450 U/L ^a^ | 1,342 (24.3) | 290 (22.9) | 310 (36.6) | 82 (10.0) | 144 (25.0) | 154 (32.2) | 25 (12.6) |
| Serum ferritin | (n = 3,185) | (n = 638) | (n = 594) | (n = 248) | (n = 427) | (n = 131) | (n = 120) |
| Median (IQR), ng/mL | 497.9 (218.7-983.4) | 482.0 (211.0-956.0) | 782.9 (399.2-1300.0) | 242.7 (111.2-461.8) | 463.0 (217.0-925.0) | 373.0 (133.0-840.0) | 350.0 (151.6-636.4) |
| 500 < ≤ 700 ng/mL ^a^ | 394 (12.4) | 84 (13.2) | 81 (13.6) | 22 (8.9) | 56 (13.1) | 15 (11.5) | 14 (11.7) |
| > 700 ng/mL ^a^ | 1,191 (37.4) | 227 (35.6) | 328 (55.2) | 38 (15.3) | 148 (34.7) | 37 (28.2) | 24 (20.0) |
| Troponin-I | (n = 1,556) | (n = 325) | (n = 469) | (n = 194) | (n = 179) | (n = 142) | (n = 39) |
| Median (IQR), ng/mL | 0.01 (0.01-0.04) | 0.01 (0.01-0.03) | 0.01 (0.01-0.02) | 0.03 (0.01-0.11) | 0.01 (0.01-0.02) | 0.13 (0.03-1.65) | 0.01 (0.01-0.01) |
| > 0.03 ng/mL ^a^ | 431 (27.7) | 85 (26.2) | 78 (16.6) | 87 (44.8) | 25 (14.0) | 101 (71.1) | < 10 ^b^ (< 25.6) |
| KL-6 | (n = 2,656) | (n = 484) | (n = 527) | (n = 286) | (n = 297) | (n = 44) | (n = 116) |
| Median (IQR), U/mL | 270.0 (199.0-432.0) | 287.0 (212.5-437.5) | 267.0 (201.0-400.0) | 401.0 (227.0-822.8) | 269.0 (194.4-449.0) | 235.5 (192.5-388.0) | 215.0 (172.0-299.0) |
| > 406 U/mL ^a^ | 736 (27.7) | 136 (28.1) | 126 (23.9) | 141 (49.3) | 89 (30.0) | 10 (22.7) | 12 (10.3) |
| Patients were categorized based on the results close to the date of first prescription (t_0_) during the period between the admission date and t_0_. | | | | | | | |
| N indicates the number of patients with a prescription pattern; n indicates the number of patients with the results of laboratory test. | | | | | | | |
| AST, aspartate aminotransferase; ALT, alanine aminotransferase; eGFR, estimated glomerular filtration; IQR, interquartile range. | | | | | | | |
| ^a^ Data are reported as No. (%). | | | | | | | |
| ^b^ When a value was less than 10, it was indicated as an aggregated value based on the MID-NET^®^ publication rule. | | | | | | | |

S1 Table. The percentage of patients with abnormal laboratory test results (continued).

|  | Ciclesonide | Casirivimab and imdevimab | Remdesivir + Dexamethasone + Baricitinib | Camostat mesylate | Favipiravir + Dexamethasone | Ivermectin | Remdesivir + Baricitinib |
| --- | --- | --- | --- | --- | --- | --- | --- |
|  | N = 126 | N = 110 | N = 107 | N = 97 | N = 96 | N = 61 | N = 55 |
| **Blood tests (biochemical tests)** | | | | | | | |
| AST | (n = 118) | (n = 67) | (n = 104) | (n = 94) | (n = 96) | (n = 58) | (n = 55) |
| Median, (IQR), IU/L | 27.0 (20.0-39.0) | 28.0 (21.0-41.0) | 46.0 (34.0-66.5) | 28.0 (21.0-42.0) | 38.5 (29.0-55.3) | 22.0 (17.0-31.0) | 66.0 (46.0-79.0) |
| ≥ 50, IU/L ^a^ | 18 (15.3) | 10 (15.0) | 46 (44.2) | 18 (19.1) | 36 (37.5) | < 10 ^b^ (< 17.2) | 39 (70.9) |
| ALT | (n = 118) | (n =67) | (n = 105) | (n = 94) | (n = 96) | (n = 58) | (n = 55) |
| Median, (IQR), IU/L | 20.5 (14.0-34.0) | 22.0 (17.0-43.0) | 33.0 (23.0-57.5) | 19.5 (12.0-47.0) | 29.3 (18.5-48.0) | 18.0 (12.0-26.0) | 50.0 (31.0-64.0) |
| ≥ 50, IU/L ^a^ | 13 (11.0) | 12 (17.9) | 36 (34.3) | 21 (22.3) | 23 (24.0) | < 10 ^b^ (< 17.2) | 28 (50.9) |
| Serum creatinine | (n = 118) | (n = 67) | (n = 105) | (n = 95) | (n = 96) | (n = 58) | (n = 55) |
| Median, (IQR), mg/dL | 0.81 (0.61-0.98) | 0.87 (0.73-1.05) | 0.82 (0.65-0.96) | 0.82 (0.67-1.17) | 0.94 (0.79-1.15) | 0.88 (0.72-1.01) | 0.88 (0.70-1.06) |
| > 2 mg/dL ^a^ | 0 (0.0) | < 10 ^b^ (< 14.9) | 0 (0.0) | < 10 ^b^ (< 10.5) | < 10 ^b^ (< 10.4) | < 10 ^b^ (< 17.2) | 0 (0.0) |
| eGFR | (n = 117) | (n = 67) | (n = 105) | (n = 95) | (n = 96) | (n = 58) | (n = 55) |
| Median, (IQR), mL/min/1.73m^2^ | 68.5 (56.8-86.0) | 67.6 (58.0-77.0) | 72.9 (57.2-86.4) | 65.7 (46.5-83.1) | 59.7 (47.7-68.3) | 71.4 (60.1-82.2) | 68.4 (54.3-84.4) |
| ≤ 60 mL/min/1.73m^2 a^ | 92 (78.6) | 61 (91.1%) | 82 (78.1) | 80 (84.2) | 91 (94.9) | 46 (79.3) | 44 (80.0) |
| **Severity-related biomarkers** | | | | | | | |
| Lymphocytes | (n = 112) | (n = 67) | (n = 91) | (n = 92) | (n = 94) | (n = 57) | (n = 48) |
| Median (IQR), % | 21.4 (14.5-27.0) | 20.9 (13.5-29.0) | 13.8 (9.1-21.0) | 17.3 (9.0-23.6) | 13.8 (9.6-19.2) | 25.9 (21.0-34.6) | 11.2 (9.2-16.3) |
| < 50 % | 112 (100.0) | 66 (98.5) | 90 (98.9) | 92 (100.0) | 93 (98.9) | 55 (96.5) | 48 (100.0) |
| D-dimer | (n = 89) | (n = 64) | (n = 105) | (n = 48) | (n = 93) | (n = 49) | (n = 54) |
| Median (IQR), µg/mL | 0.7 (0.5-1.2) | 0.5 (0.5-0.8) | 1.0 (0.7-1.4) | 1.7 (0.9-4.1) | 1.1 (0.8-1.8) | 0.7 (0.5-1.1) | 1.4 (1.2-1.8) |
| > 1.5 µg/mL ^a^ | 16 (18.0) | < 10 ^b^ (< 15.6) | 23 (21.9) | 25 (52.1) | 36 (38.7) | < 10 ^b^ (< 20.4) | 21 (38.9) |
| CRP | (n = 118) | (n = 67) | (n = 105) | (n = 94) | (n = 96) | (n = 58) | (n = 55) |
| Median (IQR), mg/dL | 2.3 (0.5-6.0) | 1.5 (0.6-3.8) | 8.4 (4.2-12.4) | 2.4 (0.8-5.9) | 6.5 (3.9-11.3) | 1.0 (0.1-3.4) | 10.4 (5.0-15.1) |
| 1-4 mg/dL ^a^ | 37 (31.4) | 25 (37.3) | 24 (22.9) | 29 (30.9) | 20 (20.8) | 15 (25.9) | 10 (18.2) |
| > 4 mg/dL ^a^ | 47 (39.8) | 15 (22.4) | 79 (75.2) | 32 (34.0) | 71 (74.0) | 14 (24.1) | 45 (81.8) |
| LDH | (n =118) | (n = 67) | (n = 104) | (n = 93) | (n = 95) | (n = 58) | (n = 55) |
| Median (IQR), U/L | 234.5 (183.0-316.0) | 218.0 (173.0-288.0) | 404.0 (342.5-488.5) | 221.0 (176.0-289.0) | 315.0 (244.0-426.0) | 205.0 (179.0-261.0) | 499.5 (401.0-590.0) |
| > 450 U/L ^a^ | < 10 ^b^ (< 8.5) | < 10 ^b^ (< 14.9) | 34 (32.7) | < 10 ^b^ (< 10.8) | 17 (17.9) | 0 (0.0) | 38 (69.1) |
| Serum ferritin | (n = 83) | (n = 48) | (n = 74) | (n = 26) | (n = 81) | (n = 46) | (n = 45) |
| Median (IQR), ng/mL | 222.3 (107.3-476.3) | 226.5 (118.0-514.9) | 697.0 (403.0-1302.0) | 168.0 (126.0-511.0) | 499.8 (285.0-788.0) | 96.0 (51.0-255.0) | 217.6 (761.0-1898.0) |
| 500 < ≤ 700 ng/mL ^a^ | < 10 ^b^ (< 12.0) | < 10 ^b^ (< 20.8) | 13 (17.6) | < 10 ^b^ (< 38.5) | 16 (19.8) | < 10 ^b^ (< 21.7) | < 10 ^b^ (< 22.2) |
| > 700 ng/mL ^a^ | 14 (16.9) | < 10 ^b^ (< 20.8) | 36 (48.6) | < 10 ^b^ (< 38.5) | 24 (29.6) | < 10 ^b^ (< 21.7) | 34 (75.6) |
| Troponin-I | (n = 12) | (n = 16) | (n = 35) | (n = 19) | (n = 26) | (n< 10 ^b^) | (n = 10) |
| Median (IQR), ng/mL | 0.01 (0.01-0.02) | 0.01 (0.00-0.02) | 0.01 (0.01-0.01) | 0.03 (0.01-0.05) | 0.01 (0.01-0.02) | 0.02 (0.01-0.17) | 0.01 (0.01-0.02) |
| > 0.03 ng/mL ^a^ | < 10 ^b^ (< 83.3) | < 10 ^b^ (< 62.5) | < 10 ^b^ (< 28.6) | < 10 ^b^ (< 52.6) | < 10 ^b^ (< 38.5) | < 10 ^b^ (< 100.0) | 0 (0.0) |
| KL-6 | (n = 79) | (n = 28) | (n = 70) | (n < 10 ^b^) | (n = 80) | (n = 43) | (n = 46) |
| Median (IQR), U/mL | 230.0 (174.0-307.0) | 223.5 (176.0-309.2) | 246.8 (190.1-369.6) | 233.4 (188.0-307.0) | 258.5 (190.0-412.1) | 203.0 (155.0-236.0) | 348.5 (247.0-433.0) |
| > 406 U/mL ^a^ | 11 (13.9) | < 10 ^b^ (< 35.7) | 15 (21.4) | < 10 ^b^ (< 100.0) | 21 (26.3) | < 10 ^b^ (< 23.3) | 15 (32.6) |
| Patients were categorized based on the results close to the date of first prescription (t_0_) during the period between the admission date and t_0_. | | | | | | | |
| N indicates the number of patients with a prescription pattern; n indicates the number of patients with the results of laboratory test. | | | | | | | |
| AST, aspartate aminotransferase; ALT, alanine aminotransferase; eGFR, estimated glomerular filtration; IQR, interquartile range. | | | | | | | |
| ^a^ Data are reported as No. (%). | | | | | | | |
| ^b^ When a value was less than 10, it was indicated as an aggregated value based on the MID-NET^®^ publication rule. | | | | | | | |
